# Quarantine-related traumatic stress, views, and experiences during the first wave of Coronavirus pandemic: A mixed-methods study among adults in Saudi Arabia

**DOI:** 10.1371/journal.pone.0261967

**Published:** 2022-01-13

**Authors:** Halah Bin Helayel, Anwar Ahmed, Syed Khabir Ahmed, Abeer Ahmad, Ruhi Khan, Samar A. Al-Swailem

**Affiliations:** King Khaled Eye Specialist Hospital, Riyadh, Saudi Arabia; Seoul National University College of Medicine, REPUBLIC OF KOREA

## Abstract

**Purpose:**

Mental health is a significant problem following exposure to a traumatic event. This study aimed to examine quarantine-related experiences, traumatic stress, and coping strategies among adults quarantined in Saudi Arabia due to coronavirus disease 2019 (COVID-19) exposure or travel history.

**Methods:**

Individuals aged ≥ 18 years who were quarantined in Saudi Arabia due to COVID-19 exposure or travel history were included. We used a sequential mixed methods design, using an online survey followed by in-depth individual telephonic interviews. The Impact of Event Scale-Revised (IES–R) was used to measure post-traumatic stress disorder (PTSD) symptoms after the quarantine. To identify factors associated with significant symptoms (IES–R score ≥ 33), prevalence ratios (PR) with 95% confidence intervals were computed using Poisson regression with robust error variance. In the next phase, a subset of the participants (n = 26) were interviewed to elicit their quarantine-related experiences and coping responses. Major themes and subthemes were identified.

**Results:**

Of the 111 adults who completed the survey, 32 (28.8% [95% CI, 21.1–38.0%]) had significant PTSD symptoms (IES–R score ≥ 33) and 27 (24.3% [95% CI, 17.2–33.3%]) had severe symptoms (IES–R score > 37). Marital status was the only variable that was significantly associated with significant PTSD symptoms (P = 0.028). Significant symptoms were twice as prevalent in married adults than among other marital groups (PR 2.00, 95% CI, 1.08–3.72). Participants reported negative emotions such as overwhelming fear, helplessness, anxiety, and disgust. Participants utilized both problem-centered coping (e.g., use of social support) and emotion-centered coping (e.g., use of positive diversionary activities) during the quarantine period.

**Conclusion:**

PTSD symptoms were present in one out of every four quarantined persons. The quarantine experience is viewed negatively. These findings highlight the need for increased awareness about stress-related disorders among quarantined individuals. Efforts are needed to detect and manage these symptoms early while making the quarantine experience more satisfying for the involved individuals and groups.

## Introduction

Coronavirus disease 19 (COVID-19) was identified in December 2019 in Wuhan, China. It causes illness ranging from the simple common cold to severe acute respiratory syndrome. The critical level of the spread and infection resulted in an outbreak characterized as a pandemic [1].

The World Health Organization (WHO) provided standardized preventive public health measures to stop or slow down its spread. Furthermore, one of the most effective measures recommended is quarantine. The Centers for Disease Control and Prevention (CDC) defined quarantine as separation and restriction of movement of people who are potentially at risk of exposure to a contagious disease to assure if they become sick or prevent them from transmitting disease [2]. Quarantine is an effective approach that might protect everyone during infectious disease outbreaks [3]. Although quarantine is the most feasible way to control disease spread during pandemics and outbreaks, most published studies found that it can also have a negative impact among individual who are quarantined. Quarantine is often described as a bad experience that causes emotional disturbance and acute stress disorders including post-traumatic stress disorder (PTSD) due to the detachment from social life and family [1, 4]. Quarantine differs from isolation or curfew, where isolation is defined as the seclusion of those afflicted with an infectious illness from healthy unaffected individuals; however, the two definitions may be used interchangeably, particularly in the media [5].

Thus, these studies have suggested that the needs of quarantined individuals should be fully addressed during this time and efforts should be made to ensure a tolerable experience [6]. Additionally, success can only be achieved by reducing the negative psychological impact of quarantine [3]. Different tools are available to assess the psychological impact of traumatic events including, the Impact of Event Scale-Revised (IES-R) and the Depression, Anxiety, and Stress Scale (DASS-21) [7–11].

On a local level, the Ministry Of Health in Saudi Arabia has applied all preventable measures to overcome this pandemic. The aim of these measures is mainly to contain the outbreak to limit disease spread. These measures include quarantine (home or a facility) for everyone returning from other countries starting from February 28, 2020.

Previous studies from Saudi Arabia were focused on the impact of the disease itself on the mental health of the general population, healthcare workers, or during the curfew period. To the best of our knowledge, this is one the first studies to assess the impact of quarantine on individuals returning from abroad or those who were exposed to confirmed cases at the beginning of the COVID-19 pandemic [12–17]. The present study specifically evaluates the immediate stress and psychological impact experienced by quarantined adults during the outbreak period of SARS-CoV-2. Data from this study can be used to help develop coping strategies and to mitigate the impact of stress. Additionally, it can help develop a future management plan that can be applied locally and worldwide to avoid short-term and long-term impacts of the pandemic on mental illness.

## Methods

**Fig 1:**
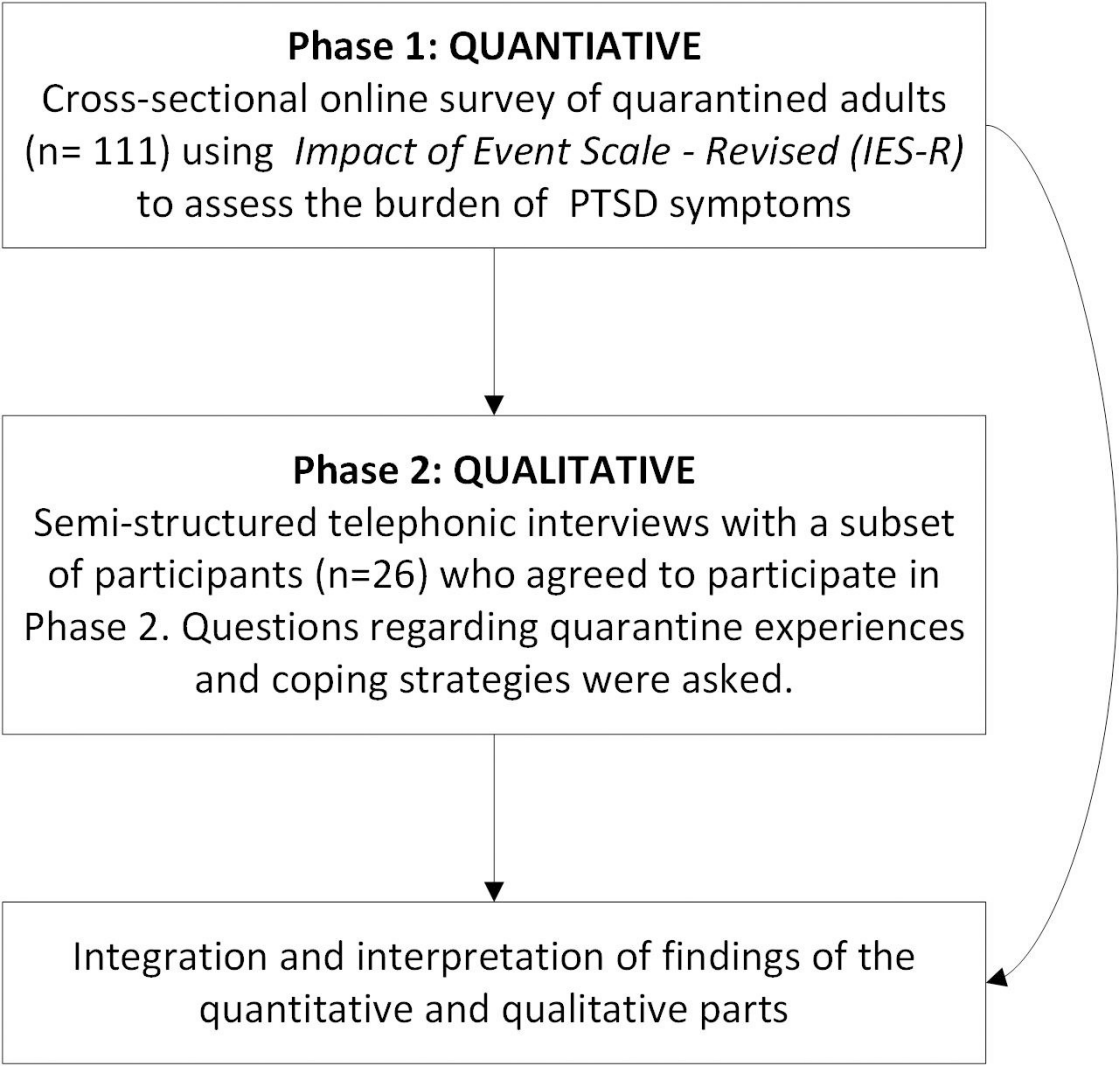
A schematic diagram of the study mixed design process.

This study focused on individuals aged ≥ 18 years who were quarantined at home, hotel or a health care facility in Saudi Arabia due to COVID-19 exposure or travel history. We included individuals who had completed their quarantine. The study was approved by the Institutional Review Board (IRB) of King Khaled Eye Specialist Hospital (KKESH, [IRB No. P-2045]) and adhered to the tenets of the Declaration of Helsinki. Informed consent was obtained from the participants. Data were collected during May 2020 to June 2020. We used a sequential mixed methods design, using an online cross sectional survey (QUAN) followed by in-depth individual telephonic interviews (QUAL). We used a convenient and snowball sampling method to select participants (Fig 1). Participation was voluntary and complete anonymity of the participants was ensured. The survey was distributed using professional social media platforms (LinkedIn) or direct invitation through text messages or WhatsApp between May 1 and June 30, 2020.

The main outcome measures were self-reported distress which was measured using the *The Impact of Event Scale-Revised* (IES–R), which is among the most widely used scales to identify PTSD symptoms [18]. IES-R has been used in previous outbreaks, such as SARS in 2003 [7].

Data were collected on the demographics and socioeconomic characteristics (age, gender, nationality, years of education, employment, monthly income, number of household members), the reason for quarantine (travel or exposure), travel (date of arrival, country visited, quarantine date), quarantine setting (house, hotel or a designated government facility), duration of quarantine, and stress. The IES-R scale was used to assess distress following quarantine [18]. We translated the scale into Arabic and back-translated into English to represent the population living in Saudi Arabia.

Study participants were asked to indicate the extent to which each item distressed or bothered them over the last 14 days using a rating scale of 0–5. The IES-R yields a total score ranging from 0 to 88. This item scale is composed of three subscales measuring avoidance, intrusion, and hyperarousal. Each item’s response is rated from 0 to 4, where “0” indicates no symptoms and “4” indicates extreme symptoms [19]. The total IES-R score is subdivided into 0–23 (normal), 24–32 (mild), 33–36 (moderate), and >37 (severe) [19]. A total score of 33 or greater is suggestive of probable PTSD [8].

In the second phase of this study, 26 of 111 participants who agreed to participate in in-depth interviews were interviewed via telephone using a semi-structured interview guide. Face-to-face interviews were not possible due to strict social distancing measures. Each participant was asked a series of questions about their quarantine experiences and coping strategies. The interviews were conducted by a trained clinical research coordinator. The interview questions were developed by the two of the authors (HBH and AA) and reviewed by the other members of the research team. The interview was approximately 20 minutes. The design, conduct, and reporting of the second phase of the study followed the consolidated criteria for reporting qualitative studies (COREQ) guidelines [20].

### Sample size

The IES-R scale contains 22 items. Our sample size was based on a ratio of five persons per item [21], which equals a minimum of 110 respondents.

### Data analysis

Quantitative data were entered into Microsoft Excel 2010 (Microsoft Corp., Redmond, WA, USA) and analyzed using STATA 16.1 (StataCorp LLC, College Station, TX, USA). Categorical data were presented as frequencies and percentages. Continuous variables were reported as means and standard deviation. An IES–R score ≥ 33 was considered indicative of probable PTSD. The determinants of probable PTSD were examined using Poisson regression with robust error variance. A correlation matrix was developed to examine the relationship among the three subscales for the IES-R: Intrusion, Avoidance and Hyperarousal.

For univariate analysis, covariates examined in relation to probable PTSD were: current age, gender, marital status, education level, nationality, employment status, and monthly income, quarantine facility type, duration of quarantine and reason for quarantine. Covariates with *P* ≤ 0.2 in the univariate analysis were evaluated using multivariable analysis. A *P* < 0.05 was considered statistically significant.

Responses to qualitative interview questions were written down verbatim and translated into English. MAXQDA 2020, ([software], Berlin, VERBI Software, 2019) was used to organize the data and identify themes. Two of the authors (HBH and AA) identified themes independently, and resolved any disagreement through discussion. Meaningful quotations were selected to represent important themes.

## Results

### Quantitative results

Table 1 presents the characteristics of the study participants. One thousand and thirty-five individuals opened the online survey by clicking on the link that was provided to them. One hundred and eleven of these individuals were eligible and completed the survey. The study sample was comprised of 48 (43.2%) males, and 63 (56.8%) females. Nearly half (47.7%) of the participants were aged 30 to 49 years. The reason for quarantine was either a history of recent travel to a high-risk country (60.4%) or exposure to a confirmed case (39.6%). Two-thirds of the cases were quarantined for 14 days or longer.

**Table 1:** Characteristics of survey participants (n = 111).

*Characteristic*	Frequency	%
**Age, years**		
<30	47	42.3
30–49	53	47.7
≥ 50	11	9.9
**Gender**		
Male	48	43.2
Female	63	56.8
**Current marital status**		
Single	63	56.8
Married	48	43.2
**Highest completed education level**		
High school	30	27.0
Higher	81	73.0
**Nationality**		
Non-Saudi	19	17.1
Saudi	92	82.9
**Current employment status**		
Employed	85	76.6
Unemployed	26	23.4
**Monthly income of the participant, SAR**		
<6000	24	21.6
≥ 6000	87	78.4
**Quarantine facility**		
Home	50	45.0
MoH Health care facility	43	38.7
Hotel	18	16.2
**Duration of quarantine, days**		
<14	27	24.3
≥ 14	84	75.7
**Reason for quarantine**		
Travel to a high-risk area	67	60.4
Exposure to a confirmed case	44	39.6

**Fig 2:**
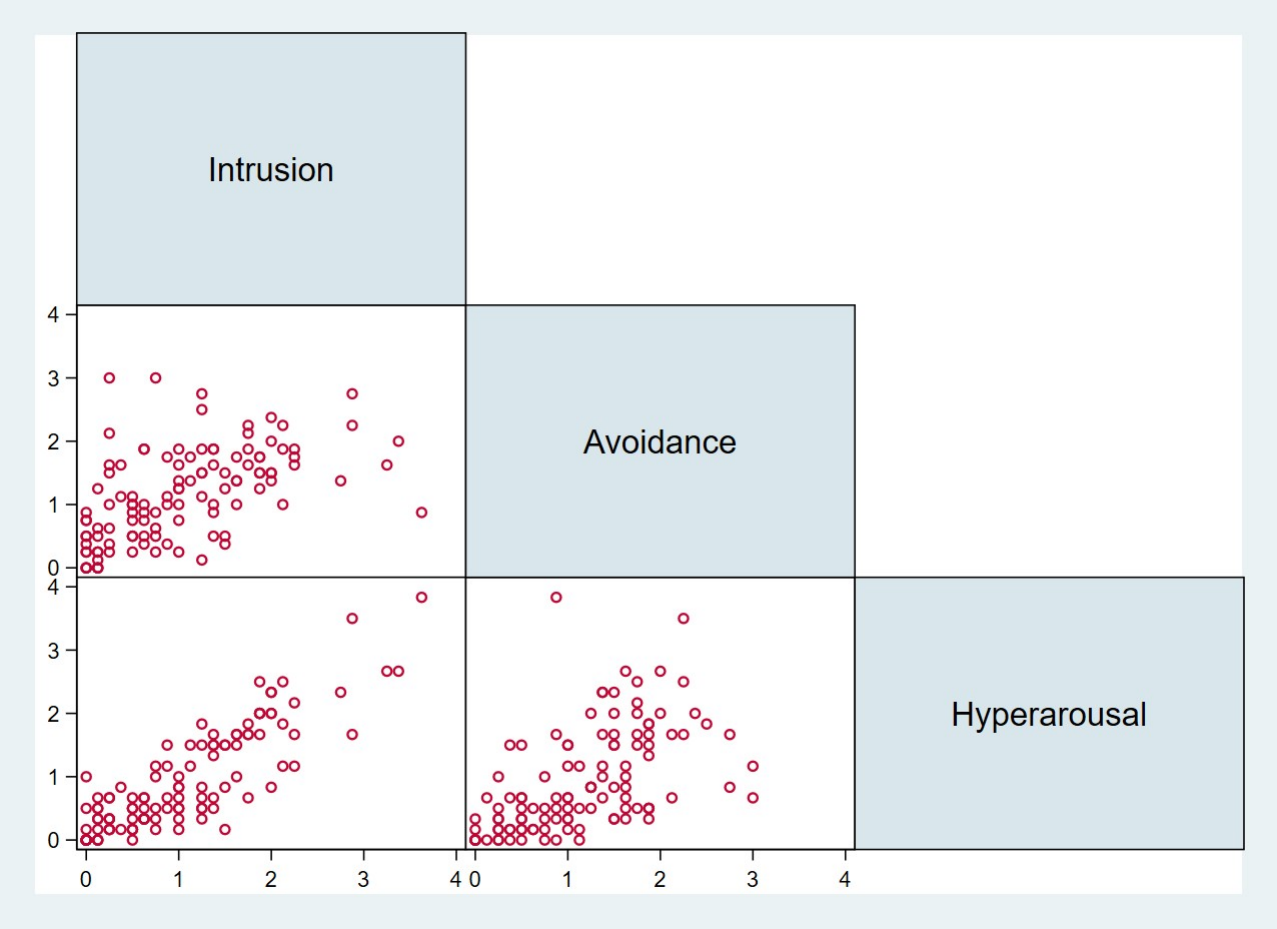
A scatter plot matrix that shows the relationship among the three subscales.

We found a high level of internal consistency for the IES-R (average inter-item covariance: 0.44, Cronbach’s alpha: 0.92, items: 22) and its subscales Intrusion (average inter-item covariance: 0.63, Cronbach’s alpha: 0.89, items: 8), Avoidance (average inter-item covariance: 0.41, Cronbach’s alpha: 0.78, items: 8) and Hyperarousal (average inter-item covariance: 0.54, Cronbach’s alpha: 0.81, items: 6). Fig 2 is a scatter plot matrix that shows the relationship among the three subscales. There was a moderate correlation between Intrusion and Avoidance (r = 0.54), and Avoidance and Hyperarousal (r = 0.55). There was a strong correlation between Intrusion and Hyperarousal (r = 0.86). The mean scores for the subscales Intrusion, Avoidance and Hyperarousal were 1.06 ± 0.84, 1.17 ± 0.72 and 0.94 ± 0.82, respectively.

Table 2 presents the frequency of probable PTSD across the study subgroups.

**Table 2:** Frequency of probable post traumatic stress disorder *–overall and across subgroups.

*Characteristic*	Interviewed	Individuals with probable PTSD
	n	Freq (%)
**All**	**111**	**32 (28.8)**
**Age, years**		
<30	47	9 (19.1)
30–49	53	18 (34.0)
≥ 50	11	5 (45.5)
**Gender**		
Male	48	15 (31.2)
Female	63	17 (27.0)
**Current marital status**		
Single	63	12 (19.0)
Married	48	20 (41.7)
**Highest completed education level**		
High school	30	5 (16.7)
Higher	81	27 (33.3)
**Nationality**		
Non-Saudi	19	5 (26.3)
Saudi	92	27 (29.3)
**Current employment status**		
Employed	85	27 (31.8)
Unemployed	26	5 (19.2)
**Monthly income of the participant, SAR**		
< 6000	24	6 (25.0)
≥ 6000	87	26 (29.9)
**Quarantine facility**		
Home	50	19 (38.0)
Health care facility (MoH)	43	11 (25.6)
Hotel	18	2 (11.1)
**Duration of quarantine, days**		
<14	27	7 (25.9)
≥ 14	84	25 (29.8)
**Reason for quarantine**		
Travel to a high-risk area	67	15 (22.4)
Exposure to a confirmed case	44	17 (38.6)

PTSD = post-traumatic stress disorder

*Probable PTSD = The Impact of Event Scale-Revised score ≥ 33

**Fig 3:**
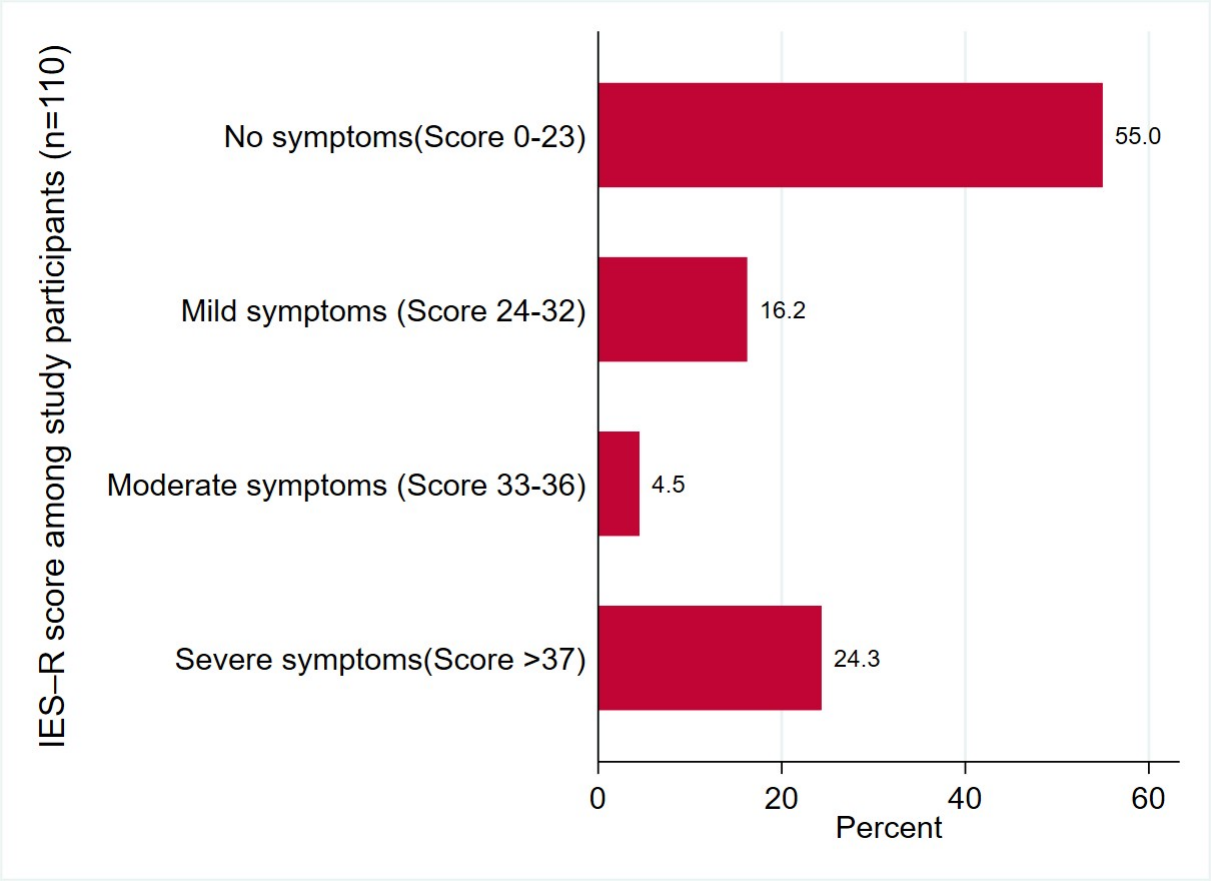
A bar chart that shows the IES-R Score among study participants.

Of the 111 adults who completed the survey, 32 (28.8% [95% CI, 21.1–38.0%]) had significant PTSD symptoms (IES–R score ≥ 33), ranging from 11.1% among those who were quarantined in hotels to 45.5% among those aged ≥ 50 years. Twenty-seven (24.3% [95% CI, 17.2–33.3%]) participants had severe symptoms (IES–R score > 37, Fig 3).

Table 3 presents the univariate and multivariable Poisson regression analysis of the determinants of probable PTSD.

**Table 3:** Factors associated with post-traumatic stress disorder among survey participants (n = 111).

*PTSD*	Univariate analysis				Multivariable analysis			
	PR*	LCI	UCI	*P*	PR	LCI	UCI	*P*
**Age, years**								
<30^a^								
30–49	1.77	0.88	3.57	0.109	1.10	0.56	2.17	0.787
≥ 50	2.37	0.99	5.71	0.054	1.22	0.50	2.96	0.658
**Gender**								
Male	1.16	0.64	2.08	0.624				
Female ^a^								
**Current marital status**								
Single ^a^								
Married	2.19	1.19	4.03	0.012	2.00	1.08	3.72	0.028
**Highest completed education level**								
High school ^a^								
Higher	2.00	0.85	4.73	0.115	1.67	0.73	3.83	0.224
**Nationality**								
Non-Saudi								
Saudi ^a^	1.12	0.49	2.53	0.794				
**Current employment status**								
Employed	1.65	0.71	3.87	0.248				
Unemployed ^a^								
**Monthly income of the participant, SAR**								
< 6000 ^a^								
≥ 6000	1.20	0.55	2.58	0.649				
**Quarantine facility**								
Home	3.42	0.88	13.32	0.076	3.14	0.82	12.03	0.095
Health care facility (MoH)	2.30	0.56	9.42	0.246	2.84	0.78	10.38	0.113
Hotel ^a^								
**Duration of quarantine (days)**								
<14 ^a^								
≥ 14	1.15	0.56	2.36	0.707				
**Reason for quarantine**								
Travel to a high-risk area ^a^								
Exposure to a confirmed case	1.73	0.96	3.09	0.067	1.23	0.64	2.37	0.532

*PR = prevalence ratio

a = reference category

In the multivariable analysis, marital status was the only variable that was significantly associated with probable PTSD (*P* = 0.028), with significant symptoms twice as prevalent in married adults than among other marital groups (PR 2.00, 95% CI, 1.08–3.72). Those who were quarantined in their homes had a three times higher burden of probable PTSD than those quarantined in hotels (PR 3.14, 95% CI, 0.82–12.03), but the difference was only marginally significant (*P* = 0.095).

### Qualitative analysis

Five key themes emerged from the qualitative analysis: fear of contracting the disease or spreading the virus, coping strategies utilized during the quarantine, knowledge about the disease, positive and negative emotions, and growth under pressure (Table 4). Most of the participants interviewed had negative emotions about the quarantine such as overwhelming fear, helplessness, anxiety, and disgust. Participants utilized both problem-centered coping (e.g., use of social support) and emotion-centered coping (e.g., use of positive diversionary activities) during the quarantine period.

**Table 4:** Themes and subthemes that emerged from the interviews of participants who were quarantined during the COVID-19 pandemic.

Theme	Subtheme	Quotations
**Fear of contracting the disease or spreading the virus**		“I was afraid of transmitting the virus to my family or others.”
		“I was afraid of transmitting the infection to others, and this is what makes me always cautious and [I] rarely go out of the apartment to avoid contact with others.”
		“I may share my feelings for the first time after I came out from the quarantine, I felt great fear, and I was afraid of touching anything or dealing with anything. Whenever I go out, I feel I want to go back home as if it is my safe bubble.”
		“My biggest concern is not to get infected again.”
**Coping strategies during the quarantine**	Seeking support and experience sharing	“I was talking with a friend who was also in quarantine.”
		“I shared my experience, especially with my friends who returned with me from abroad.”
		“ Yes, I talked to those who are also in quarantine, especially to my colleague, we used to give words of encouragement to [each other] to become strong and have positive thinking, and I did not pass a day without her asking how she was”
	Adjustment	“I was reading my favorite books, watching movies and some entertainment programs, and finally sitting with my family while keeping a safe distance.”
		“I was doing physical exercises, communicating with family, watching the news, and completing work on Ph.D. research.”
		“I just accepted the idea of quarantine before I returned to Saudi Arabia. I was almost in home quarantine for more than 80 days. The idea of ​​accepting it made it go faster and easier.”
		“I spent my day with my routine from my first day to last day by starting with prayer, do deep breathing exercises, Zumba, physical exercise and dancing then video call with my husband and we eat our breakfast together, do online prayers, listen to music therapy and watch movies.”
		“I tried to focus on my health and my exams. I tried to avoid thinking about the virus and anxiety. I contacted my family and friends to avoid boredom.”
		“I Provided all my needs as much as possible. In addition to finding activities that do not require going out.”
**Knowledge about the disease and the reason for quarantine**		“The quarantine is necessary for anyone coming from abroad.”
		“I absolutely understand why I have to be quarantined … to prevent the spread of the disease (COVID-19)”
**Source of information**		“The [social media] accounts of the Ministry of Health.”
		“The news and social media.”
		“Covid-19 news was on all social media, and everyone around me was talking about it, either friends or family.”
**Positive and negative emotions**	Positive emotion	“Overall, my quarantine experience was particularly good. From the moment I was picked up from the airport until the last day of quarantine, I received an overwhelming support, and my work facility took care of my needs during the whole period, from providing foods, daily necessities, entertainment, to enquiring about my health.”
		“ I returned with an excellent feeling and performed my work with diligence.”
		“ I was very happy with the results of the swab. It was an incredibly beautiful feeling.”
		“[I] became more aware of my health.”
	Negative emotion	“Some people dealt with us like criminals.”
		“While in quarantine, I was afraid that my colleagues would not talk to me or avoid me, but I was wrong. Most of them welcomed me back.”
		“The hard thing for me was to observe myself for any symptoms. Sometimes, I imagined those with severe respiratory problems to the extent that they had to undergo intubation or be put on ventilators.”
		“Scary experience and I hope no one will try it.”
		“It was a strange experience.”
		“It was a difficult and exhausting experience, essential, and it must be accepted.”
		“It was a bad experience.”
		“I felt extreme isolation and boredom.”
		“A lack of any activity was stressful.”
		“ Fear and loneliness.”
		“Frustration at the beginning.”
		“ The experience made me somehow tense … I was worried about my children … I hope I will not go back again.”
		“Staying in one place is not a problem for me, but the presence in a room between four walls with limited space was the challenge for me.”
		“ Everything was OK except that I could not step a foot outside my apartment.”
		“ The only challenge for me was the Place because you feel like you are in prison.”
		“By transferring me to the home quarantine, the biggest challenge was to keep a social distance from my family, knowing that I had not seen them for more than ten months.”
		“Commitment to the social distance with family members, especially my mother.”
**Growth under pressure**		“A good and new experience.”
		“Truthfully, I needed a period like this. I used to feel that we previously ran continuously without dealing with life in all the details, which makes me feel that I am in a race with life, so this experience helped me calm and relaxed.”
		“A unique experience and I think it was an opportunity for the person to be alone with him/herself, whom most of the time did not find this opportunity because of commitments with family and friends, and it was also an opportunity to complete my academic duties as I am a doctoral researcher …”

## Discussion

People’s perception of stressful events may impact their mental health status, especially in difficult events such as pandemics. This mixed-methods study observed a high prevalence of probable PTSD in adults after the end of their quarantine. Quarantine experience was associated with negative emotions such as the fear of contracting the disease, the fear of spreading the virus to others and being separated from loved ones.

The psychological impact of stressful events related to infectious disease outbreaks can last for many years after these events. In one study conducted among hospital employees in Beijing three years following a SARS outbreak, it was reported that around 10% of the employees still suffered from SARS-related PTS symptoms [22]. Similarly, the majority of the disaster survivors, who experienced PTSD immediately after the disaster, were found to have such symptoms one year later [23, 24]. Lee et al [25] reported that around 80% of the population were afraid of contracting the diseases during the MERS outbreak, and 46% were found to be emotionally distressed.

During the current pandemic, Duan and Zhu [26] reported increased mental health disorders, including depression and fears. After the SARS outbreak in 2003, around 20% of the population reported being distressed [27]. In a study in Saudi Arabia [15], around 23.6% of the general public reported moderate or severe psychological impact of the COVID-19 pandemic. The same study also found that 28.3% of the study participants had moderate to severe symptoms of depression and that about 24% and 22.3% of them had anxiety and stress symptoms, respectively.

The current study showed that 28.8% of the study participants had moderate to severe PTSD symptoms (IES–R score ≥ 33). This prevalence is much higher than that reported in a recent study among medical students (n = 309) in Riyadh [28]. The study carried out during June-August 2020 found that 8.6% of the students had probable PTSD (IES–R score ≥ 33).

In a study in South Korea, the prevalence of anxiety symptoms and anger in individuals isolated during the MERS epidemic at isolation period was 7.6% and 16.6%, respectively [9]. The study used the 7-item Generalized Anxiety Disorder Scale [GAD-7]) and State-Trait Anger Expression Inventory (STAXI) to measure these disorders. The same study suggested that the progression of anxiety and anger becoming a chronic problem such as PTSD can be prevented by early and appropriate mental health care interventions.

During the SARS outbreak, certain characteristics were associated with PTSD and predicted the severity of PTS symptoms [15, 18, 29]. These included female gender, low income, duration of quarantine, exposure to a confirmed case, knowing someone infected with SARS and hospitalized. In a study on quarantined horse owners due to the equine influenza outbreak in Australia, lower education and younger age (under 24 years) were associated with a negative psychological impact [30]. In our study, marital status was the only variable that was significantly associated with probable PTSD. Married adults compared with other marital groups had a significantly higher prevalence of probable PTSD. It could be that being married may mean more duties and responsibilities, and worries about children and loss of job and livelihood. Brooks et al’s recent review highlighted the importance of communication with family and friends to relieve stress and anxiety during the quarantine period [3]. In addition to reducing boredom during the quarantine, communication with family and friends allows quarantined individuals to update their loved ones about their health and reassure them that they are well. Additionally, the presence of support groups for quarantined people is highly important. Finding peers who have been through the same situation allows for reassurance, validation, and empowerment [31]. In the second phase of our study, half of the group reported having had shared their quarantine experiences with their friends who were also in the quarantine.

Participants in our study knew the reason for their quarantine. This is contrary to previous studies which have reported insufficient knowledge of, and confusion about, the purpose of quarantine among different quarantined groups [31–36]. Our study concurs with recent findings from Saudi Arabia that highlights the role of Saudi Ministry of Health in providing timely and appropriate public information on COVID-19 [15].

The interviews provided us an in-depth understanding of the quarantine experience. Those who were quarantined described it as an unpleasant and difficult experience. The majority reported that they were afraid of contracting the disease and spreading it to their families. This finding concurs with the published literature [9, 18, 32, 36–38]. Similar to previous studies, some participants said they were particularly worried about any physical symptoms potentially related to COVID-19. Finally, some participants felt relief and happiness and reported that this experience increased their knowledge and awareness of their health.

This study has some limitations, including its cross-sectional design with no comparison group. Therefore, causal inference cannot be made with regard to the link between quarantine and significant PTSD symptoms. It is possible that in some of the study participants PTSD symptoms existed prior to quarantine, which may contaminate the interpretation of our results. Several important prequarantine predictors of psychological impact could not be assessed. This included history of psychiatric illness which has been previously shown to be associated with experiencing anxiety and anger after release from quarantine [3, 9]. We also did not inquire whether quarantined individuals have had enough supplies for their daily basic needs. Most participants spent time on social media and the internet during their quarantine; however, we did not enquire about the content that they were browsing. It is unclear as to how it impacted their mental health. Our study had a relatively small sample size, and was not a true representative of the entire quarantined population in Saudi Arabia. Our survey did not include adolescents and children due to the challenges associated with taking informed consent from these groups.

We used both the English and Arabic versions of the IES-R scale, a validated tool used to predict the presence of PTS symptoms only [7, 11]. This tool can be useful to compare the findings of different studies. The current study was an online survey that may have limited the participation of individuals with no access to internet, such as the elderly. We conducted the survey and the interview during the beginning (which was the peak) of the pandemic. We believe this might have exaggerated the impact of the quarantine on the mental health of our study participants especially during the concomitant curfew in the country and unavailability of vaccines. Therefore, prospective studies are needed to provide more reliable and long-term data to help plan strategies to improve and support the mental health of individuals subjected to quarantine.

## Conclusions

This study observed a high prevalence of probable PTSD in adults after the end of their quarantine. The quarantine experience is unpleasant. Most of this study participants expressed a wide range of negative emotions, including fear, anxiety, and frustration. Therefore, our findings highlight the importance of addressing and monitoring mental health during infectious disease outbreaks, especially among quarantined individuals. The global response to the outbreak must prioritize both the prevention and detection of psychological disorders. Additionally, research evaluating the psychological consequences of outbreaks and quarantine is highly needed to improve health services, detection, and treatment of mental health conditions.
