# Correction: Article Title: Quarantine-related traumatic stress, views, and experiences during the first wave of Coronavirus pandemic: A mixed-methods study among adults in Saudi Arabia

**DOI:** 10.1371/journal.pone.0295890

**Published:** 2023-12-11

**Authors:** Halah Bin Helayel, Anwar Ahmed, Khabir Ahmad, Abeer Ahmad, Ruhi Khan, Samar A. Al-Swailem

The third author’s name is misspelt. The correct spelling is: Khabir Ahmad. The correct citation is: Bin Helayel H, Ahmed A, Ahmad K, Ahmad A, Khan R, Al-Swailem SA (2022) Quarantine-related traumatic stress, views, and experiences during the first wave of Coronavirus pandemic: A mixed-methods study among adults in Saudi Arabia. PloS ONE 17(1): e0261967. https://doi.org/10.1371/journal.pone.0261967
